# Predictors of new-onset heart failure and overall survival in metastatic breast cancer patients treated with liposomal doxorubicin

**DOI:** 10.1038/s41598-020-75614-4

**Published:** 2020-10-28

**Authors:** Sebastian Szmit, Aleksandra Grela-Wojewoda, Małgorzata Talerczyk, Joanna Kufel-Grabowska, Joanna Streb, Jolanta Smok-Kalwat, Dariusz Iżycki, Ewa Chmielowska, Michał Wilk, Barbara Sosnowska-Pasiarska

**Affiliations:** 1 Department of Pulmonary Circulation, Thromboembolic Diseases and Cardiology, Centre of Postgraduate Medical Education, European Health Centre, Borowa 14/18, 05-400 Otwock, Poland; 2 Clinic of Clinical Oncology, Maria Sklodowska-Curie National Research Institute of Oncology, Cracow Branch, Kraków, Poland; 3 Department of Oncology, West Pomeranian Oncology Center, Szczecin, Poland; 4 Chemotherapy Ward, The Greater Poland Cancer Center, Poznan, Poland; 5 Department of Electroradiology, Poznań University of Medical Sciences, Poznan, Poland; 6 Department of Oncology, Jagiellonian University Medical College, Kraków, Poland; 7 Department of Clinical Oncology, Holy Cross Cancer Center, Kielce, Poland; 8 Department of Cancer Immunology, Poznan University of Medical Sciences, Poznan, Poland; 9 Department of Oncology, Oncology Centre, Bydgoszcz, Poland; 10 Department of Clinical Oncology, Oncologic Hospital, Tomaszów Mazowiecki, Poland; 11 Department of Oncology, Centre of Postgraduate Medical Education, European Health Centre, Otwock, Poland; 12 Department of Oncocardiology, Holycross Cancer Center, Kielce, Poland

**Keywords:** Cardiology, Oncology, Risk factors

## Abstract

Cardiovascular diseases (CVDs) are the major cause of morbidity/mortality among breast cancer (BC) patients. Observation of the daily practice in eight experienced Polish oncology centers was conducted to find all possible predictors of new cases of heart failure (HF) and overall survival (OS) of metastatic BC patients treated with liposomal doxorubicin, taking into account the impact of pre-existing CVDs. HF was the cause of premature discontinuation of liposomal doxorubicin therapy in 13 (3.2%) of 402 patients. The probability of developing HF was higher in women with pre-existing CVDs (HR 4.61; 95%CI 1.38–15.38). Independent of CVDs history, a lower risk of HF was observed in those treated with a cumulative dose of liposomal doxorubicin > 300 mg/m2 (HR 0.14; 95% CI 0.04–0.54) and taxane-naive (HR 0.26; 95% CI 0.07–0.96). Multivariate analysis including the presence of pre-existing CVDs and occurrence of new HF, revealed a liposomal doxorubicin in cumulative doses of > 300 mg/m2 as a beneficial predictor for OS (HR 0.61; 95% CI 0.47–0.78) independently of subsequent chemotherapy (HR 0.72; 95% CI 0.57–0.92) or endocrine therapy (HR 0.65; 95% CI 0.49–0.87). Higher doses of liposomal doxorubicin can decrease mortality in metastatic BC without increasing the risk of HF. The clinical benefit is achieved regardless of pre-existing CVDs and subsequent anticancer therapy.

## Introduction

Patients with breast cancer (BC) are particularly vulnerable to the cardiotoxicity of antineoplastic treatment (hormone therapy, radiation, chemotherapy, anti-HER2 drugs) and therefore the (neo-) adjuvant treatment may have an impact on the later deterioration of cardiac function and overall performance status^1,2^. Planning cancer treatments in patients with significant risk factors for developing heart failure (HF) is a major problem^3^. It is also relatively difficult to treat elderly patients or those with cardiovascular diseases (CVDs), i.e. heart disease or at least hypertension^4,5^.

Some patients with metastatic BC can live for many years and it is worth identifying clinical features that have an effect on survival. The prognosis and thus the choice of treatment can be influenced by many factors, like the hormone/HER2 receptor status, the effectiveness and toxicity of previous therapies, the patient's biological age and comorbidities. Anthracycline or taxane-based regimens are one of the preferred therapies for HER2-negative metastatic BC. Two liposomal forms of anthracyclines (pegylated and non-pegylated doxorubicin) are perceived as less toxic for the heart. The European Society for Medical Oncology (ESMO) guidelines recommend the use of liposomal doxorubicin in patients previously treated (adjuvant or palliative therapy) with conventional anthracyclines or taxanes and who do not require complex chemotherapy (I/A). Based on this document, liposomal doxorubicin may also be an option in subsequent lines of anticancer treatment even in HER2-positive metastatic BC (II/A)^6^.

The presented study based on the daily practice of eight experienced Polish oncology centers has been undertaken to check the real impact of liposomal doxorubicin on cardio-oncology prognoses of metastatic BC patients.. Two main purposes were defined.

- *cardiology purpose* identification of predictors of new-onset HF adjusted for the presence of pre-existing CVDs.
- *oncology purpose* assessment of how the administration of liposomal doxorubicin can influence overall survival (OS).

The final aim in the study was to assess OS in multivariate analysis including the history of pre-existing CVDs, the occurrence of new-onset HF,, patients' demographic characteristics (age, obesity etc.), characteristics of cancer disease as well as characteristics of therapy with liposomal doxorubicin, previous and subsequent anticancer therapy.

## Results

Retrospective analysis covered 402 women with metastatic BC. Characteristics of histopathological diagnoses, localization of metastases and previous antitumor treatment are presented in detail in Table 1.

**Table 1:** Characteristics of all 402 patients in relationships with presence of cardiovascular diseases (CVDs).

Evaluated factors		All 402 patients N (%)	Patients with cardiovascular diseases N = 141 (%)	Patients without cardiovascular diseases N = 261 (%)	*p* value
Clasical CV risk factors	Diabetes	20 (5.0)	18 (12.8)	2 (0.8)	< 0.0001
	Obesity (BMI ≥ 30 kg/m^2^)	83 (20.6)	42 (29.8)	41 (15.7)	0.0009
	Older age (≥ 60y)	185 (46)	91 (64.5)	94 (36)	< 0.000001
	Hypothyroidism	17 (4.2)	11 (7.8)	6 (2.3)	0.009
Histopatological diagnosis	ER or PR positive	331 (82.3)	116 (82.3)	215 (82.4)	0.98
	HER2 positive	77 (19.2)	32 (22.7)	45 (17.2)	0.18
	Triple negative	48 (11.9)	13 (9.2)	35 (13.4)	0.22
Site of metastases	Pericardium / myocardium	4 (1)	2 (1.4)	2 (0.8)	0.92
	Lung	135 (33.6)	56 (39.7)	79 (30.3)	0.056
	Pleura	38 (9.5)	16 (11.3)	22 (8.4)	0.34
	Liver	179 (44.5)	59 (41.8)	120 (46)	0.43
	Bone	185 (46)	63 (44.7)	122 (46.7)	0.69
	Nodules	98 (24.4)	39 (27.7)	59 (22.6)	0.26
	Soft tissue or skin	45 (11.2)	21 (14.9)	24 (9.2)	0.08
	Central nervous system	36 (9)	11 (7.8)	25 (9.6)	0.55
	Peritoneal metastases	17 (4.2)	5 (3.5)	12 (4.6)	0.81
	Ovary / uterus	12 (3)	5 (3.5)	7 (2.7)	0.86
	Local progression	33 (8.2)	7 (5.0)	26 (10)	0.08
Previous adjuvant anticancer therapy	Radiotherapy: left-sided	101 (25.1)	32 (22.7)	69 (26.4)	0.41
	Radiotherapy: right-sided	126 (31.3)	45 (31.9)	81 (31)	0.86
	Doxorubicin in adjuvant setting	166 (41.3)	53 (37.6)	113 (43.3)	0.27
	Taxanes in adjuvant setting	74 (18.4)	33 (23.4)	41 (15.8)	0.057
	Anti-HER2 in adjuvant setting	19 (4.7)	10 (7.1)	9 (3.4)	0.1
	Endocrine therapy in adjuvant setting	64 (15.9)	27 (19.1)	37 (14.2)	0.19
Previous neoadjuvant anticancer therapy	No neoadjuvant	305 (75.9)	113 (80.1)	192 (73.6)	0.14
	Doxorubicin in neoadjuvant setting	96 (23.9)	28 (19.9)	68 (26.1)	0.16
	Taxanes in neoadjuvant setting	68 (16.9)	17 (12.1)	51 (19.5)	0.056
	Doxorubicin and Taxanes in neoadjuvant setting	67 (16.7)	17 (12.1)	50 (19.2)	0.068
Previous palliative anticancer therapy	Doxorubicin in palliative setting	46 (11.4)	15 (10.6)	31 (11.9)	0.71
	Taxanes in palliative setting	97 (24.1)	31 (22)	66 (25.3)	0.46
	Anti-HER2 in palliative setting	33 (8.2)	13 (9.2)	20 (7.7)	0.59
	Endocrine therapy in palliative setting	74 (18.4)	29 (20.6)	45 (17.2)	0.41
Previous treatment with conventional doxorubicin	Conventional doxorubicin dose ≥ 250 mg/m2	170 (42.3)	51 (36.2)	119 (45.6)	0.068
	Conventional doxorubicin dose ≥ 400 mg/m2	26 (6.5)	6 (4.3)	20 (7.7)	0.18
Taxane-naive		190 (47.3)	70 (49.6)	120 (46)	0.48
Anthracycline—naive		119 (29.6)	51 (36.2)	68 (26.1)	0.03
Characteristics of therapy with liposomal doxorubicin	First line in palliative setting	234 (58.2)	82 (58.2)	152 (58.2)	0.99
	Cumulative dose > 300 mg/m2	158 (39.3)	53 (37.6)	105 (40.2)	0.6
	Monotherapy	82 (20.4)	28 (19.9)	54 (20.7)	0.84
	Treatment time > 3.5 months	202 (50.2)	67 (47.5)	135 (51.7)	0.42
	Treatment time ≤ 2 months	95 (23.6)	39 (27.7)	56 (21.5)	0.16
	Cardiovascular events as end of treatment	14 (3.5)	8 (5.7)	6 (2.3)	0.078
	Heart failure as end of treatment	13 (3.2)	8 (5.7)	5 (1.9)	0.08
	Hematological toxicity as end of treatment	20 (5)	6 (4.3)	14 (5.4)	0.63
	Other toxicity as end of treatment	12 (3)	8 (5.7)	4 (1.5)	0.04
	PFS > 5 months	196 (48.8)	64 (45.4)	132 (50.6)	0.32
Subsequent anticancer therapy	Cytostatics	189 (47.1)	68 (48.2)	121 (46.4)	0.72
	Taxanes	93 (23.1)	27 (19.1)	66 (25.3)	0.18
	Endocrine therapy	129 (32.1)	47 (33.3)	82 (31.4)	0.69

Most of the women had received conventional doxorubicin treatment in the past: the median dose was 240 mg/m2, (interquartile range, IQ: 0–360 mg/m2). CVDs as well as their significant risk factors were frequent (Table 1). Among those studied, patients with metastatic BC and CVDs more often had concomitant diabetes, obesity, hypothyroidism and were older (≥ 60y). Although there were no differences in the histological type, the location of metastases and indications for adjuvant / neoadjuvant therapy, patients with a history of CVDs were more likely to avoid conventional anthracyclines at an earlier stage of cancer therapy 51/36.2% vs 68/26.1% (*p* = 0.03).

The median duration of active liposomal doxorubicin treatment was 106 days (3.5 months). The median total dose of liposomal doxorubicin of 300 mg/m2, (IQ:180–360 mg/m2) was given. The median number of cycles of liposomal doxorubicin was six.

Out of 402 patients, in 230 women (57.21%), the treatment was terminated due to cancer progression. Moreover, 46 women (11.44%) had the treatment withdrawn due to unacceptable toxicity: 20 women (4.98%) had a hematological toxicity, 14 women experienced cardiovascular complications (3.5%), including 13 cases of new-onset HF with reduced ejection fraction (3.2%) and one case of acute pulmonary embolism (Table 1). In each case of HF, the left ventricular ejection fraction was significantly reduced, i.e. below 40% when their baseline level had been above 50%.

In addition to hematological and cardiac toxicity, 12 women (3%) were diagnosed with other serious toxicity: general poor treatment tolerance (n = 4), significant deterioration of general condition and weakness (n = 3), hepatotoxicity (n = 2), acute infection (n = 1), unacceptable skin complications (n = 1), hypersensitivity reaction (n = 1).

The remaining 126 women (31.34%) completed liposomal doxorubicin treatment based on the decision of an independent oncologist. Each of these patients was under clinical observation to assess the onset of their cancer progression.

Factors predicting the occurrence of new-onset HF during active liposomal doxorubicin treatment were (Table 2): pre-existing CVDs HR 4.61 (95% CI 1.38–15.38, *p* = 0.01), previous treatment with taxanes HR 3.76 (95% CI 1.03–13.73; *p* = 0.04) especially taxanes in a palliative setting HR 3.5 (95% CI 1.13–10.9; *p* = 0.03), and a cumulative dose of liposomal doxorubicin > 300 mg/m2: HR 0.16 (95% CI 0.04–0.6; *p* = 0.007). Previous conventional doxorubicin therapy in a palliative setting was of borderline significance (HR 3.23; 95% CI 0.87–12.02; *p* = 0.08) even with a cumulative dose ≥ 400 mg/m2 (HR 4.26; 95% CI 0.91–19.79; *p* = 0.06). Regardless of CVDs history, a lower risk of HF was observed in patients treated with cumulative doses of liposomal doxorubicin > 300 mg/m2 in comparison to others (HR 0.14; 95% CI 0.04–0.54; *p* = 0.004). A higher risk of new-onset HF was observed for patients after previous palliative chemotherapy with taxanes (HR 3.85; 95% CI 1.23–12.02; *p* = 0.02), or conventional doxorubicin (HR 3.48; 95% CI 0.93–12.95; *p* = 0.06) especially in doses ≥ 400 mg/m2 (HR 4.07; 95% CI 0.88–18.93; *p* = 0.07).

**Table 2:** Predictors of new-onset heart failure (time to heart failure during active treatment with liposomal doxorubicin).

Evaluated factors		Univariate analysis			Analysis adjusted for presence of CVDs		
		HR	95%CI	*p* value	HR	95%CI	p-value
Patients with cardiovascular diseases (CVDs) vs patients without CVDs		4.61	1.38–15.38	0.01	–	–	–
Older age (≥ 60y vs < 60y)		1.16	0.37–3.6	0.8	–	–	–
Histopatological diagnosis	ER or PR positive	1.82	0.23–14.25	0.57	–	–	–
	HER2 positive	1.05	0.29–3.84	0.94	–	–	–
	Triple negative	0.9	0.11–7.02	0.92	–	–	–
Previous adjuvant anticancer therapy	Radiotherapy: left-sided	0.54	0.12–2.43	0.42	–	–	–
	Radiotherapy: right-sided	1.27	0.4–3.98	0.68	–	–	–
	Doxorubicin in adjuvant setting	0.88	0.28–2.8	0.83	–	–	–
	Taxanes in adjuvant setting	1.23	0.34–4.5	0.75	–	–	–
	Anti-HER2 in adjuvant setting	0.91	0.11–7.56	0.93	–	–	–
	Endocrine therapy in adjuvant setting	0.45	0.06–3.52	0.45	–	–	–
Previous neoadjuvant anticancer therapy	Doxorubicin in neoadjuvant setting	1.44	0.39–5.38	0.58	–	–	–
	Taxanes in neoadjuvant setting	1.35	0.29–6.22	0.7	–	–	–
Previous palliative anticancer therapy	Doxorubicin in palliative setting	3.23	0.87–12.02	0.08	3.48	0.93–12.95	0.06
	Taxanes in palliative setting	3.5	1.13–10.9	0.03	3.85	1.23–12.02	0.02
	Anti-HER2 in palliative setting	1.17	0.15–9.08	0.88	–	–	–
	Endocrine therapy in palliative setting	2.3	0.69–7.67	0.17	–	–	–
Previous treatment with conventional doxorubicin	Conventional doxorubicin dose ≥ 250 mg/m2 vs < 250 mg/m2	1.58	0.53–4.71	0.41	–	–	–
	Conventional doxorubicin dose ≥ 400 mg/m2 vs < 400 mg/m2	4.26	0.91–19.79	0.06	4.07	0.88–18.93	0.07
Taxane-naive vs pretreated with taxanes		0.27	0.07–0.97	0.04	0.26	0.07–0.96	0.04
Anthracycline—naive vs pretreated with anthracyclines		0.69	0.19–2.57	0.58	–	–	–
Characteristics of therapy with liposomal doxorubicin	First vs next line of palliative setting	0.68	0.22–2.12	0.51	–	–	–
	Cumulative dose > 300 mg/m2 vs ≤ 300 mg/m2	0.16	0.04–0.6	0.007	0.14	0.04–0.54	0.004
	Monotherapy vs combination with cyclophosphamide	1.16	0.35–3.85	0.8	–	–	–

The median of progression-free survival (PFS) was 150 days (5 months). During the observation 306 patients (76,1%) died. The median OS was 492 days (16.4 months). Factors significantly affecting OS were: ER or PR positive disease (HR 0.71; 95% CI 0.54–0.95; *p* = 0.02) or HER2 positive (HR 1.38; 95% CI 1.06–1.82; *p* = 0.02), metastases localization: liver (HR 1.33; 95% CI 1.06–1.66, *p* = 0.01) or central nervous system (HR 1.64; 95% CI 1.15–2.35; *p* = 0.006), no neoadjuvant therapy (HR 0.65; 95% CI 0.5–0.83, *p* = 0.0007), doxorubicin and taxanes in neoadjuvant setting (HR 1.46; 95% CI 1.1–1.94; *p* = 0.008) as well as earlier palliative treatment with taxanes (HR 1.57; 95% CI 1.22–2.04; *p* = 0.0005), anti-HER2 (HR 1.5; 95% CI 1.02–2.2; *p* = 0.04) or endocrine therapy (HR 1.58; 95% CI 1.2–2.1; *p* = 0.001). Liposomal doxorubicin treatment had a positive effect on OS if the following conditions were met: first line of palliative setting (HR 0.53; 95% CI 0.42–0.66; *p* < 0.000001), cumulative dose > 300 mg/m2 (HR 0.53; 95% CI 0.42–0.67; *p* < 0.000001), treatment duration > 3.5 months (HR 0.48; 95% CI 0.38–0.61; *p* < 0.000001). Patient’s characteristics with longer OS included being taxane-naive (HR 0.57; 95% CI 0.46–0.72; *p* = 0.000002) and anthracycline-naive (HR 0.63; 95% CI 0.48–0.81; *p* = 0.0004).

In Table 3, the key cardio-oncology predictors of OS were presented in multivariate analysis. Independent of the presence of pre-existing CVDs and occurrence of HF: liposomal doxorubicin in cumulative dose > 300 mg/m2 (HR 0.61; 95% CI 0.47–0.78; *p* = 0.0001)*,* subsequent chemotherapy (HR 0.72; 95% CI 0.57–0.92; *p* = 0.008) or endocrine therapy (HR 0.65; 95% CI 0.49–0.87; *p* = 0.003) were confirmed as significant and positive. There was borderline significance from previous conventional doxorubicin doses ≥ 250 mg/m2 (HR 0.75; 95% CI 0.56–1.00; *p* = 0.05).

**Table 3:** The selected cardio-oncology predictors of overall survival.

Evaluated factors		Univariate analysis			Multivariate analysis		
		HR	95%CI	*p* value	HR	95%CI	*p* value
Patients with pre-existing cardiovascular diseases (CVDs) vs patients without CVDs		0.999	0.79–1.27	0.996	0.998	0.77–1.29	0,99
Patients with heart failure as end of treatment with liposomal doxorubicin vs patients without this complication		1.31	0.73–2.33	0.36	1.08	0.59–1.98	0.8
Older age (≥ 60y vs < 60y)		1.04	0.83–1.30	0.76	1.19	0.93–1.53	0.17
Obesity (BMI ≥ 30 kg/m^2^ vs < 30 kg/m^2^)		0.76	0.57–1.01	0.054	0.85	0.63–1.14	0.28
Prognostically unbeneficial site of metastases	Central nervous system vs other sites	1.64	1.15–2.35	0.006	1.5	1.02–2.18	0.04
	Liver vs other sites	1.33	1.06–1.66	0.01	1.43	1.12–1.83	0.004
	Pericardium / myocardium vs other sites	2.25	0.83–6.06	0.11	2.06	0.74–5.76	0.17
Histopatological diagnosis of breast cancer	HER2 positive vs other diagnosis	1.38	1.06–1.82	0.02	1.28	0.95–1.73	0.11
	Triple negative vs other diagnosis	1.28	0.92–1.79	0.14	1.15	0.8–1.66	0.45
Taxane-naive vs pretreated with taxanes		0.57	0.46–0.72	0.000002	0.8	0.59–1.07	0.14
Anthracycline—naive vs pretreated with anthracyclines		0.63	0.48–0.81	0.0004	0.79	0.55–1.14	0.21
Patients after left-sided radiotherapy vs others		1.27	0.99–1.64	0.06	1.02	0.78–1.33	0.9
Patients treated earlier with conventional doxorubicin dose ≥ 250 mg/m2 vs others		1.17	0.93–1.46	0.18	0.75	0.56–1.00	0.05
Characteristics of therapy with liposomal doxorubicin	First vs next line ofpalliative setting	0.53	0.42–0.66	< 0.000001	0.82	0.61–1.11	0.2
	Monotherapy vs combination with cyclophosphamide	1.31	0.996–1.74	0.05	1.01	0.74–1.39	0.9
	Cumulative dose > 300 mg/m2 vs ≤ 300 mg/m2	0.53	0.42–0.67	< 0.000001	0.61	0.47–0.78	0.0001
Subsequent anticancer therapy	Patients treated with cytostatics vs others	0.96	0.77–1.2	0.71	0.72	0.57–0.92	0.008
	Patients treated with endocrine therapy vs others	0.56	0.44–0.72	0.000007	0.65	0.49–0.87	0.003

## Discussion

Metastatic BC remains a disease with an unfavorable prognosis, in which the median survival is about 3 years and only 25% of patients survive 5 years^7,8^. The problem is its heterogeneity, incomprehensible mechanisms of resistance to various therapies and the lack of predictive factors in many clinical situations. An additional issue is the coexistence of significant chronic comorbidities such as CVDs^9^. In our study, patients with a history of CVDs, despite the higher risk of developing HF, did not have shorter OS rates. We believe, that thanks to the guideline-guided treatment of HF with reduced ejection fraction, our patients did not die prematurely. The recognized new HF episodes did not affect the benefits of liposomal doxorubicin treatment.

A large percentage of women with breast cancer experience a relapse after radical treatment. In an era when anthracyclines and taxanes are widely used in adjuvant treatment, the choice of optimal treatment for metastatic BC takes on a new dimension especially with respect to anthracycline reuse^10–12^. In terms of cardiac safety, it seems justified to use liposomal doxorubicin after previous adjuvant treatment in its conventional form^13–15^. However, analysis of four prospective studies on pegylated liposomal doxorubicin in metastatic BC showed that taxane naïve patients or those with a better ECOG functional class have the greatest clinical benefit and thus significantly longer survival rates^16^. The previous dose of conventional anthracyclines, however, was of little importance^17^. The univariate analysis of our study confirms these regularities: the greatest benefit for survival is obtained by patients without prior treatment with taxanes or anthracyclines. On the other hand, it should be admitted that the previous use of conventional doxorubicin in doses ≥ 250 mg/m2 may have positive significance for OS, which was observed in multivariate analysis of 18 cardio-oncology predictors. Furthermore, surprisingly, in univariate analysis adjusted for the presence of CVDs, previous conventional anthracyclines treatment even in high doses (≥ 400 mg/m2) was of borderline relevance to the risk of HF development during liposomal doxorubicin therapy, while taxanes were of greater importance.

In a scientific discussion, the use of liposomal doxorubicin as maintenance therapy was also considered. While this is not possible with conventional doxorubicin due to the risk of cumulative dose-dependent cardiotoxicity, with liposomal doxorubicin this problem is significantly reduced^18^. Our observations indirectly confirm this possibility, especially since longer OS was obtained at higher doses of liposomal doxorubicin (above 300 mg/m2) and when this treatment lasted longer than 3.5 months. However, further research is needed to determine the optimal duration of such therapy and optimal dosing of liposomal doxorubicin per cycle.

There are a number of common risk factors for developing both BC and CVDs, including diabetes, hypertension, hyperlipidemia, and obesity^19,20^. Compared to the general population, breast cancer patients also have an increased cardiovascular mortality, this risk is about twice as high when considering age, menopausal status and other classic risk factors^21^. Recent data suggest that women with a history of coronary artery disease, hypertension or diabetes have a significantly increased risk of cardiovascular complications during both adjuvant and palliative treatment for BC^22^. Polish data indicate that the occurrence of HF in patients with metastatic BC is a consequence of previous (neo) adjuvant chemotherapy with anthracyclines and a history of coronary events^23^. It becomes clear that the increased cardiovascular morbidity in women with BC is due to the frequent occurrence of adverse classic risk factors that are often not optimally controlled (e.g. smoking, low physical activity, high BMI, lipid disorders) and adverse effects of cancer treatment^24,25^. Left breast radiotherapy compared to right breast radiotherapy is also associated with a higher risk of cardiovascular death as well as death from myocardial infarct^26^. The risk of cardiovascular death is higher after anthracycline treatment for breast cancer if women had other risk factors such as diabetes or hypertension^27,28^. Interestingly, in our study main classic risk factors like obesity (with probably insulin resistance) or older age were more common in patients with CVDs, but they were not associated with higher risk of mortality in univariate and multivariate analysis.

The cumulative dose is the strongest risk factor for anthracycline cardiotoxicity^29^. The risk increases with the administered dose, but there is no safe dose of anthracyclines. Everything depends on one’s individual predisposition. Cardiovascular events and symptomatic HF were observed at lower doses (< 400 mg/m2) especially among patients with cardiovascular risk factors^30^. Elderly patients with concomitant heart or vascular diseases and those receiving other cytostatics with synergistic cardiotoxicity potential are at greatest risk. An important finding of our Polish study is the lack of influence of high cumulative doses of liposomal doxorubicin on the occurrence of HF. Interestingly, we confirmed the importance of other anti-cancer drugs, primarily widely used taxanes. In our opinion, accurate cardiological screening of patients receiving taxanes is needed, especially if patients have a history of concomitant CVDs. Anthracyclines are avoided in these patients, but taxanes are widely used, which is also confirmed by our Polish observations.

The mission of cardio-oncology should be a full personalization of the treatment, i.e. a collective, simultaneous look at both cardiological and oncological problems, which can be the cause of either premature death or worsening of a patient’s quality of life. A special and rare challenge of cardio-oncology is the treatment of diseases when cancer directly involves the structure of the cardiovascular system^31,32^. A more frequent task of cardio-oncology is an antitumor treatment of patients with a history of CVDs or those experiencing new iatrogenic cardiovascular complications during oncological treatment^33,34^. It is suggested that HF induced by conventional anthracycline therapy is associated with a risk of increased mortality^35,36^. Our Polish research confirms the successful use of liposomal doxorubicin even in patients with metastases to myocardium/pericardium or after previous therapy with conventional doxorubicin. This study demonstrates that the new cases of HF induced by liposomal doxorubicin were not associated with a significant increase in mortality.

The optimal cardiovascular protection in oncology that the cardiologist can use is a debatable issue^37^. There are trials confirming or contradicting the role of beta-blockers and inhibitors of the renin–angiotensin–aldosterone system (RAAS) in the prevention of primary anthracycline cardiotoxicity, especially in patients without classic risk factors for HF. On the other hand, it should be stated that people treated with beta-blockers or RAAS inhibitors due to CVDs are more likely to experience HF^38^. This is undoubtedly a consequence of the main risk factor in this case being hypertension or coronary artery disease, treated with a beta-blocker or RAAS inhibitors. Our study confirmed CVDs as the main predictors for the development of HF even in patients treated with liposomal doxorubicin.

Various prevention strategies are undertaken in the field of clinical oncology^39^. The calculation of the cumulative anthracycline dose seems to be a special concern^40^. It has been confirmed that higher cumulative doses of liposomal doxorubicin (pegylated or nonpegylated) are associated with a significantly lower risk of cardiotoxicity in comparison to conventional anthracyclines (OR = 0.18; 95% CI, 0.08–0.38)^41^. Both liposomal forms are available in the treatment of metastatic BC, and they are considered as effective as conventional doxorubicin, and definitely safer for the heart^42^. Observations of the Polish Lymphoma Research Group show that it is possible to use liposomal doxorubicin safely and beneficially for the course of treatment in patients with co-existing CVDs^43^. Although we treat older and more cardiologically ill patients, thanks to the use of liposomal doxorubicin the prognosis can be even better^44^.

The American Society of Clinical Oncology (ASCO)^45^ indicates the use of liposomal forms of doxorubicin in patients who are to receive high doses of anthracyclines (e.g. doxorubicin ≥ 250 mg / m2) as one of the preventive strategies for reducing the risk of cardiovascular complications. The European Society of Cardiology (ESC) in its position paper on the toxic effect of oncological treatment on the cardiovascular system^46^ confirms that statement. The document also emphasizes that choosing liposomal doxorubicin maintains efficacy comparable to conventional anthracycline. Our Polish results from daily practice support clearly these opinions from ASCO and ESC.

Our study has several limitations. First, its retrospective design may lead to data inconsistency. There was insufficient control over the principles of the initially used cardiac treatment for CVDs. Secondly, we analyzed a heterogenic population of metastatic BC patients (e.g. receptor status, number of metastatic sites etc.). In addition, there were substantial differences in previous and subsequent treatment lines. This could have had an important impact on survival statistics. Thirdly, patients were receiving one of two liposomal doxorubicin forms understood as comparable. Despite these limitations, to our knowledge, this study is the world’s largest summary of clinical experience with liposomal doxorubicin treatment regarding predictors of new-onset heart failure and overall survival in metastatic BC patients. We can conclude that in patients with metastatic BC, prolonged liposomal doxorubicin treatment, i.e. lasting more than 3.5 months, and thus the administration of a higher cumulative dose, i.e. above 300 mg/m2, significantly increases OS. Although ESMO guidelines recommend the administration of liposomal doxorubicin after taxane or conventional anthracycline therapy, our univariate analysis demonstrates that the longest OS can be seen in patients who had not previously received these drugs. However, it should be noted that, prior treatment involving conventional doxorubicin does not shorten OS. The greatest benefit may be achieved among patients with positive hormone receptors, when subsequent therapy was possible and effective independent of pre-existing CVDs and new HF. However, BC patients triple negative or HER2 positive did not have worse OS based on the multivariate analysis of 18 cardio-oncology predictors.

Concomitant CVDs are obviously associated with a higher risk of the occurrence of HF during liposomal doxorubicin treatment and should also be carefully evaluated in this population^47^. Notwithstanding this, patients with metastatic BC and concomitant CVDs may not have a worse prognosis, because newly diagnosed HF does not have to increase the overall mortality. It is worth considering that the earlier therapy with taxanes is a risk factor for developing HF.

Further research is needed to define a better clinical position for liposomal anthracyclines.

## Methods

The study provides a retrospective summary of data on metastatic BC treatment in eight Polish oncology centers: the beginning of therapy in the period from March 2008 to September 2018 (10.5 years), with the closing of follow-up in August 2019. In each case an oncologist independently recognized that the metastatic BC patient should benefit from liposomal doxorubicin treatment and qualified for this therapy for three important reasons: (1) the predicted high efficacy of doxorubicin in metastatic BC; (2) co-existing cardiovascular diseases (CVDs); (3) previous treatment with conventional anthracyclines. Two liposomal forms of doxorubicin are available in Europe for metastatic BC: (1) pegylated liposomal doxorubicin indicated as a monotherapy in patients at increased risk of cardiac complications, (2) non-pegylated liposomal doxorubicin indicated as first-line treatment, used in combination with cyclophosphamide.

In the analysis of the risk of HF and cardio-oncology predictors of mortality as the primary goal, the following factors were taken into account:

- medical history, including pre-existing CVDs, metabolic diseases such as diabetes and obesity and unmodifiable risk factors such as age and gender,
- hormone/HER2 receptor status, metastasis location,
- previous neoadjuvant, adjuvant, palliative treatment with special focus on the dose of conventional doxorubicin
- duration and cumulative dose of liposomal doxorubicin, observed toxicity including cardiovascular and hematological complications, obtained progression-free survival time (PFS)
- subsequent anticancer therapy lines after liposomal doxorubicin.

The study was performed in accordance with the local bioethics committee guidelines. Pre-existing CVDs should be confirmed earlier by independent certified cardiologists and treated according to guidelines^48^. Patients received angiotensin-converting enzyme inhibitors (ACEIs) or angiotensin II receptor blockers (ARBs) due to arterial hypertension. Statins were used for hypercholesterolemia management. Beta-blockers were continued because of earlier recognized coronary artery disease or arrhythmias. Exclusion criteria were defined as: all types of heart failure previously recognized, even with preserved ejection fraction (EF), all types of asymptomatic left ventricular dysfunction with ejection fraction below 50%, and each hemodynamicly important heart valve disease. Patients with breast cancer metastases to pericardium or myocardium could be included.

In our hypothesis HF with reduced ejection fraction below 40% may be the real reason for terminating cancer therapy and can have a significant impact on the overall survival of patients with metastatic BC^49^. The definition of HF with reduced ejection fraction proposed by the European Society of Cardiology was adopted in the study^50^. In each case of such HF the appropriate guideline-guided treatment was used, including beta-blockers, RAAS inhibitors etc.

Statistical analysis was performed by Statistica (StatSoft), nominal parameters were presented as a percentage frequency, comparisons between groups with and without CVDs were made using the chi^2 test with Yates correction if indicated. Survival curves and Cox proportional hazard model were used to determine predictors for the occurrence of HF during active liposomal doxorubicin treatment in univariate analysis, then the most significant results were adjusted for the presence of CVDs. Correlations between selected cardio-oncology variables and mortality risk were performed in the univariate and multivariate Cox proportional hazard analysis. The results of the Cox models were presented as hazard ratios and 95% confidence interval (CI), a *p* value of less than 0.05 was considered significant.

### Ethical approval

The protocol of the study was approved by Bioethical Committees at the Centre of Postgraduate Medical Education in Poland (Resolution Number 16/PB/2020) and was in accordance with the 1964 Helsinki declaration and its later amendments or comparable ethical standards.

### Informed consent

Written informed consent of the treatment with liposomal doxorubicin was obtained from all patients.
